# Design and Implementation of an Indoor Warning System with Physiological Signal Monitoring for People Isolated at Home

**DOI:** 10.3390/s22020590

**Published:** 2022-01-13

**Authors:** Chi-Huang Hung, Yong-Yi Fanjiang, Yi-Shiune Lee, Yi-Chao Wu

**Affiliations:** 1 Department of Information Technology, Lee-Ming Institute of Technology, New Taipei City 24346, Taiwan; allen@mail.lit.edu.tw; 2 Graduate Institute of Applied Science and Engineering, Fu Jen Catholic University, New Taipei City 242062, Taiwan; 407068021@gapp.fju.edu.tw; 3 Department of Computer Science and Information Engineering, Fu Jen Catholic University, New Taipei City 242062, Taiwan; 4 Interdisciplinary Program of Green and Information Technology, National Taitung University, Taitung 95092, Taiwan; alanwu@nttu.edu.tw

**Keywords:** Bluetooth beacon, indoor position, physiological signal monitoring

## Abstract

Due to the recent COVID-19 pandemic, many people have faced in-home isolation, as every suspected patient must stay at home. The behavior of such isolated people needs to be monitored to ensure that they are staying at home. Using a camera is a very practical method. However, smart bracelets are more convenient when personal privacy is a concern or when the blood oxygen value or heart rate must be monitored. In this study, a low-cost indoor positioning system that uses a Bluetooth beacon, a smart bracelet, and an embedded system is proposed. In addition to monitoring whether a person living alone is active in a specific environment and tracking the heart rate or blood oxygen value under particular conditions, this system can also send early warning signals to specific observation units or relatives through instant messaging software.

## 1. Introduction

In this era of rapid technological advancement, Internet of Things technology not only allows remote control of equipment but also provides various daily life assistance services. Regarding the application of this technology in the family setting, the most frequently discussed research topics are accident prevention for elderly people, physiological monitoring of sick patients, home safety, and other topics. In the context of fall prevention for elderly people, some researchers have asked elderly individuals to wear various sensors, such as accelerometers, gyroscopes, and magnetometers/electronic compasses, to monitor balance, classify and recognize fall postures, and determine whether the individuals were likely to fall [1]. Fall detection has also been applied in other situations. For example, one study investigated the installation of devices on construction personnel to obtain physiological data and environmental data. The aim was to prevent solitary construction workers from being injured without being discovered, which causes more serious injury [2]. Daily life tracking can be used to assess the health condition of a person being cared for by medical staff. A study proposed a method to detect the strength of a beacon signal in the room through a smartphone. This system collects the time spent by an elderly individual in each room, as well as relevant physiological parameters. Medical staff analyze changes in this information to understand the patient’s personal habits [3,4]. Another study investigated the installation of a Beacon receiver in the room to detect a Bluetooth device on the user’s body. The researchers used the signal strength value between two devices, calculated the distance, and analyzed which room had the highest utilization rate [5]. The user’s location information can allow rescue units to quickly reach their destination. The most commonly used positioning technology is the global positioning system (GPS). However, GPS can only be used for outdoor positioning and cannot provide effective location information for the interior home environment. Therefore, many studies have proposed different indoor positioning methods to overcome the challenge of indoor location data collection. One study proposed a method to calculate the user’s location using the difference in Wi-Fi signal strength between a smart watch and the router [6].

Modern smart watches can record physiological information such as pace, heartbeat, and blood oxygen, and they use a Wi-Fi connection to make data transmission more convenient. Diversified data types allow researchers to analyze user behavior patterns more effectively. Many studies have proposed other indoor positioning methods to reduce the cost of deploying a large number of Wi-Fi wireless base stations and used RFID, Bluetooth, or ZigBee, which have low-cost equipment, as wireless communication methods for in-door positioning. To solve these problems, one research group proposed a positioning system using hierarchical classification. This system divides the environment into multiple regions to reduce the number of training and prediction categories for the classifier. The benefit of this design is that it provides a high-precision positioning system for large-scale environments. However, this approach has a higher cost than other methods, and it is not suitable for a family environment [7]. Some studies have proposed the use of multiple microcontrollers with Bluetooth modules for indoor positioning. By deploying a large number of low-cost microcontrollers and Bluetooth modules, the accuracy of the data can be improved [8]. A Bluetooth module can be used for indoor positioning. In one article, the investigators replaced the Bluetooth module with a Bluetooth Low Energy (BLE) module that has lower power consumption and can provide a more stable and effective signal source and a long equipment operation time [9,10]. The BLE Beacon method is used for indoor positioning and equipment control. The received signal strength indication (RSSI) data from multipoint beacon receivers are collected for triangulation. They can be used to analyze users’ locations and control appliances in the surrounding area [11,12]. Alternatively, such systems can use Beacon technology with an inertial measurement unit (IMU) to record the user’s historical movement trajectory [13].

In recent years, countries worldwide have attached great importance to human rights and personal privacy. In the past, a simple method to improve or monitor the home environment through Internet of Things technology was to use a photographic lens for environmental recording or behavior monitoring. However, some spaces or conditions, such as changing rooms, toilets, and VIP rooms, are not suitable for solving problems with this method. In light of continued progress in the development of Beacon indoor positioning technology, an increasing number of studies have used this method to analyze the user’s location and provide related services. One study used Beacon and smartphones to locate indoor positions in a museum, analyzed the locations of visitors, and provided related cultural content to visitors near works of art [14]. In this method, a customized application program must be executed on a smartphone to provide a complete service. This makes this approach inconvenient for users who do not have a smartphone or have not installed the customized application program. Some studies have focused on deploying multipoint Beacon receivers to receive BLE beacons or smartphone signals from users. An article reported the integration of Beacon positioning technology and smartphones in the context of a parking system, with parking guidance services provided to users through the system [15].

In other studies, related technologies have been applied to campus personnel management. Investigators detected smart Bluetooth devices on students and transportation vehicles to determine which vehicles students should take and to indicate whether all individuals who took the school bus arrived at their destination. When a situation occurs, the teacher or the driver can be notified through instant messaging software [16]. Other research institutes have applied this technology to detect the attendance status of students in a class to build an automatic absence detection and record system. Teachers can use the time typically spent on manual attendance for more effective academic activities, such as quizzes and discussions [17]. Although the indoor positioning technology proposed above provides information for the research team to estimate the user’s location, Beacon uses the 2.4 GHz frequency band to transmit data. This frequency band is a public band, which means that many signals will cause interference. In addition, the accuracy of the RSSI value is easily affected. Many studies have proposed different methods, such as filtering, numerical averaging, other algorithms, and deep learning, to improve the accuracy of Beacon indoor positioning [18,19,20,21,22,23].

Sensor data collection is the most important part of these systems, but how to access the collected data is an important factor for data analysis. The processing performance of many IoT devices is limited by the available resources in terms of computing power and network transmission. For data transmission between devices, at the same bandwidth transmission speed, the size and format of the data content will affect the transmission time and the accuracy of the data transmission. Compared with the hypertext transfer protocol (HTTP), the convenience of the message queuing telemetry transport (MQTT) protocol opens up new applications for the development of the IoT industry. In one study, MQTT agreements were used to realize the concept of home Internet of Things services. This approach uses a topic-based publish/subscribe model and converts messages into tween devices. Each device is a client and communicates with and controls specific topics on the proxy server [24].

**Figure 1:**
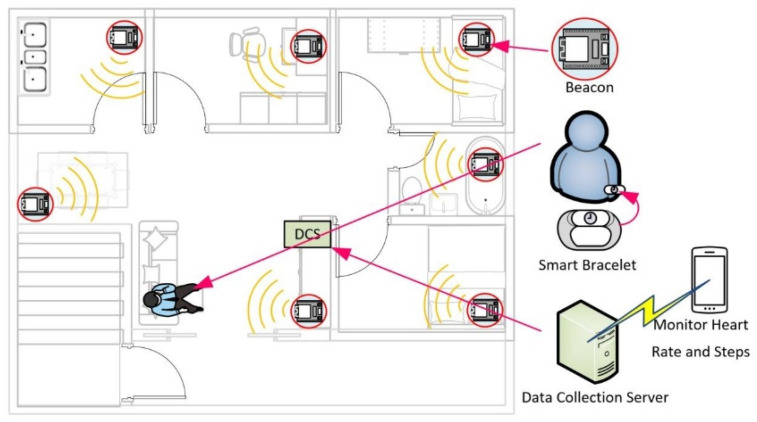
Configuration diagram of the low-cost indoor positioning system.

Currently, there is an increasing number of people living alone. In addition, some specific individuals need to be temporarily quarantined due to COVID-19. These individuals can receive accommodation in designated hotels, however, in some cases they will be quarantined at home on designated premises. Therefore, this paper proposes a low-power indoor monitoring system that can be quickly installed in a specific space or room, as shown in Figure 1. This paper offers the following ways to improve the situation that may occur in the isolation monitoring process:

1. To confirm whether quarantined individuals are staying in the designated room according to the regulations, they can be monitored through card swipe record of access control or telecommunication signal of a smartphone. However, similar methods cannot confirm whether the user is truly staying in the designated room. This study proposes to detect the user’s location through Bluetooth beacons scattered throughout the room. The Bluetooth beacon detects the user’s smart bracelet and determines the user’s location based on the strength of the Bluetooth signal it reads. If a user’s position does not change for a long time, a monitoring unit can be provided to determine whether the user stays in the room.
2. To accurately monitor users’ activities, the simplest way is to use cameras to watch them, but this method may infringe on users’ privacy. In this study, in addition to reading the Bluetooth signal strength to determine the user’s position, the position level of the user’s smart bracelet is also analyzed through the Bluetooth signal strength. When the position or height of the bracelet exceeds the specified range, the user may have abnormal conditions. The smart bracelet worn by the user can also perform some simple physiological measurements and determine whether the user needs medical assistance based on the measured data.
3. Quarantine is usually temporary or short-term residence and is unsuitable for complex monitoring systems in isolation rooms. Therefore, a Bluetooth beacon device with low power consumption and wireless transmission is adopted in this study. It is only necessary to arrange several beacon devices in the designated room, install smart bracelets on users’ arms, and a data collection server in the room. Our proposed method not only can be quickly installed but can also be quickly dismantled and recycled after isolation for use in other spaces.

## 2. Materials and Methods

Currently, there are many techniques for indoor positioning. Each technology has its pros, cons, and the context in which it is applicable. This section briefly introduces commonly used indoor positioning technologies on the market and compares their advantages and disadvantages shown in Table 1.

### 2.1. Discussion on Indoor Positioning Technology

#### 2.1.1. Radio Frequency Identification (RFID) Indoor Positioning Technology

RFID is the modulation of radio signals to electromagnetic fields by radio frequency (RF) mode [25,26]. The tag attached to the item generates an induced current after passing through the magnetic field to transmit the data to achieve the purpose of identification and triangulation multi-pair two-way communication. RFID indoor positioning technology has a short range of actions and a wider transmission range due to the non-line sight electromagnetic field. The size of the logo is relatively small, and the cost is relatively low. However, it lacks communication capabilities, is less resistant to interference, is not easily integrated into other systems, and has poor user security and privacy protection and international standardization.

#### 2.1.2. Wi-Fi Positioning Technology

There are two types of Wi-Fi positioning technology [27,28]. One is to triangulate a person or vehicle more accurately using a differential algorithm by using the wireless signal strength of a mobile device and three wireless network access points. Another approach is to prerecord the signal strength of many identified anchor points and determine the location by comparing the signal strength of the new device with an extensive database of data. Wi-Fi positioning can achieve complex and large-scale positioning, monitoring, and tracking tasks, with a wide range of applications, but the indoor positioning accuracy can only reach approximately 2 m. Due to the popularity of Wi-Fi routers and mobile terminals, location systems can share the network with other customers. The hardware cost is meager, and Wi-Fi location systems can reduce the possibility of RF interference.

#### 2.1.3. Ultra-Wideband (UWB) Positioning Technology 

UWB technology is a new wireless communication technology that has emerged in recent years and is very different from traditional communication technology [29,30]. Compared to conventional communication systems, it does not use carriers but transmits data by sending and receiving very narrow pulses in nanoseconds or microseconds and thus has a bandwidth of 3.1~10.6 GHz.

UWB technology is wireless with a high transmission rate, low transmission power, and high penetration power. It is based on very narrow pulses and carrierless waves that make it possible to obtain more accurate results in the field of indoor positioning. However, the disadvantage is that peripheral devices are not yet widespread.

#### 2.1.4. Ultrasonic Positioning Technology 

Ultrasonic positioning technology is achieved by installing multiple ultrasonic speakers in a room and detecting ultrasonic signals through terminal microphones for indoor positioning [31,32]. The location of the terminal device can be estimated by the difference in arrival time of different sound waves. The wireless synchronization of this system can be done very simply by sending a signal through the ultrasonic transmitter, which is then received by the microphone on the receiver and then calculated by the system for positioning. The relatively slow propagation of sound waves takes a rather long time to transmit the same content. Greater system capacity can only be achieved by the time difference of arrival (TDoA) method.

#### 2.1.5. ZigBee Wireless Network Technology 

ZigBee is a new short-range, low-rate wireless network technology [33,34]. These sensors require very little power consumption and transmit data via a cascade of radio waves from one node to another. ZigBee works very efficiently as a low-power communication system. Multipath effects and movement greatly influence ZigBee signal transmission. In addition, the positioning accuracy depends on the physical quality of the signal, the density of the signal source, the environment, and the effectiveness of the algorithm, which also leads to higher software costs.

#### 2.1.6. Infrared 

Infrared waves are electromagnetic waves with wavelengths between radio waves and visible light waves. Infrared indoor positioning technology is based on the principle that modulated infrared light is emitted by an infrared transmitter and received by an optical sensor installed in the room for positioning [35,36]. Although infrared is more accurate in indoor positioning, it can only travel within the line of sight because light cannot pass through obstacles. The two main disadvantages of line of sight and short transmission distance make the indoor positioning effect very poor. It will not work correctly when the logo is in a pocket or has a wall or other cover. Reception antennas need to be installed in every room and corridor, which is expensive. Therefore, infrared is only suitable for short-distance transmission, is prone to interference from fluorescent lamps or indoor lights and has limitations in precise positioning.

#### 2.1.7. Bluetooth Technology 

Bluetooth technology measures signal strength for location and is a wireless transmission technology with short distances and low power consumption [37,38]. The positioning can be obtained by installing several suitable Bluetooth LAN access points in the room, configuring the network as a multiuser-based network connection, and ensuring that the Bluetooth LAN access point is always the master device of the piconet. Bluetooth technology is mainly used to locate small areas, such as single-layer lobbies or warehouses. The most significant advantage of Bluetooth indoor location technology is that it is small and easy to integrate into PDA, PC, and smartphones, so it is easy to popularize. It is easy to find equipment, and signal transmission is not affected by visual distance when using this technique for indoor short-distance positioning. Depending on the technology or algorithm used by different companies, the accuracy can be kept at 3–15 m.

### 2.2. Hardware Architecture

**Figure 2:**
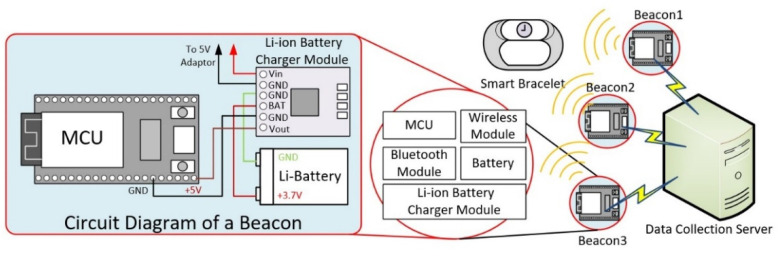
Hardware architecture and circuit diagram of the low-cost indoor positioning system.

The hardware architecture of the proposed system consists of Bluetooth beacons, smart bracelets, and data collection servers, as shown in Figure 2.

#### 2.2.1. Bluetooth Beacon

The Bluetooth beacon comprises a microcontroller (MCU), a Bluetooth module, a wireless network module, and a lithium battery charging module. The ESP32 chip is used in the microcontroller, including Bluetooth and wireless networking capabilities. In addition to reducing the cost of purchasing additional modules, the integrated design also reduces the size of the device. The circuit diagram is shown on the left side of Figure 2. It detects the signal strength between smart bracelets through the Bluetooth module and uses a media access control address (MAC address) to ensure the uniqueness of detected bracelets. Each beacon has a lithium battery and charging circuit or is powered by an external power adapter to provide a stable power supply over time.

#### 2.2.2. Smart Bracelet

The primary function of the smart bracelet in the proposed approach is to send a Beacon signal so that the Bluetooth Beacon can search the devices and provide essential physiological measurement functions in specific situations. The bracelets can be divided into self-designed bracelets and commercial bracelets. The self-designed bracelet contains a microcontroller with an ESP32 core chip, a MAX30100 heart rate monitoring module, a lithium battery, and a charging module and can send data via Wi-Fi. The second model is for commercial use and uses a miniature embedded system that measures heart rate and sends data via Bluetooth.

#### 2.2.3. Data Collection Server

The data collection server uses Raspberry Pi, a miniature single board computer. The system uses the ARM architecture and contains 2–8 GB of main memory, system storage by TF card, USB interface, HDMI video output, RCA terminal output, and built-in Ethernet/WLAN/Bluetooth network linking. The device can be used to develop interactive products. For example, it can read many switch and sensor signals and control various physical devices, such as lights and electronic components. Instead of using a traditional computer or cloud computing server, the system uses an embedded system as a data acquisition system (DCS). For example, it can read many switch and sensor signals and control various physical devices, such as lights and electronic components [39,40,41]. This model reduces the cost and allows data to be stored only in the data acquisition system; therefore, there is no need to worry about compromising data security during network transmission. The system organizes and manages RSSI data from various beacons through the built-in WLAN service. In addition, important information can also be sent over the internet to designated external systems or organizations via the built-in Ethernet or an additional USB network card installed.

### 2.3. Software Components

**Figure 3:**
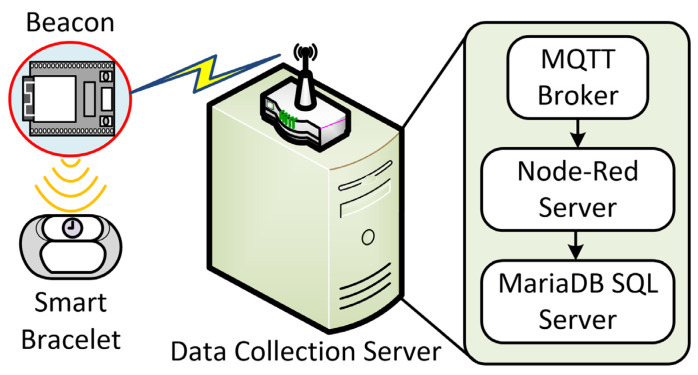
The software architecture of DSC.

Figure 3 shows the software architecture of the data collection server. The data collection server is the primary data integration center of the system, including providing data transmission, data management, and data storage services. Each of these services is detailed below.

#### 2.3.1. Data Transmission

Message queuing telemetry transport (MQTT) is used for data transmission between Bluetooth beacons and data acquisition systems. The system provides a Message Broker service. Each sensor and data collection server is regarded as a single client. The beacons can send RSSI values to the specified topics by posting and subscribing. The Data Acquisition Server subscribes to the selected topic. The subscriber receives the data messages published by the beacon from each topic and stores them in the data management tool.

#### 2.3.2. Data Management

The system uses Node-Red as a management tool to connect the data sent by the subscribed topics to the database for collection and storage. Node-Red is a visual development tool developed based on Node.js. It can be used with many modules for rapid development and various implementations. It is a graphical interface management program often used in Internet of Things technology. The system installed MQTT, HTTP, MySQL, instant messaging software, and other modules in Node-Red tools through the above modules and other services for data exchange. The set program stores the data in the database or starts the specified message notification service.

#### 2.3.3. Data Storage

The system uses MariaDB as the database management system because it is a replica of MySQL, an open-source database, and the primary database supported by Raspberry Pi. The name, time, date, RSSI signal strength value, and system time of the DCS device will be stored in the MariaDB database through the Node-Red management tool.

### 2.4. Experimental Methods

**Figure 4:**
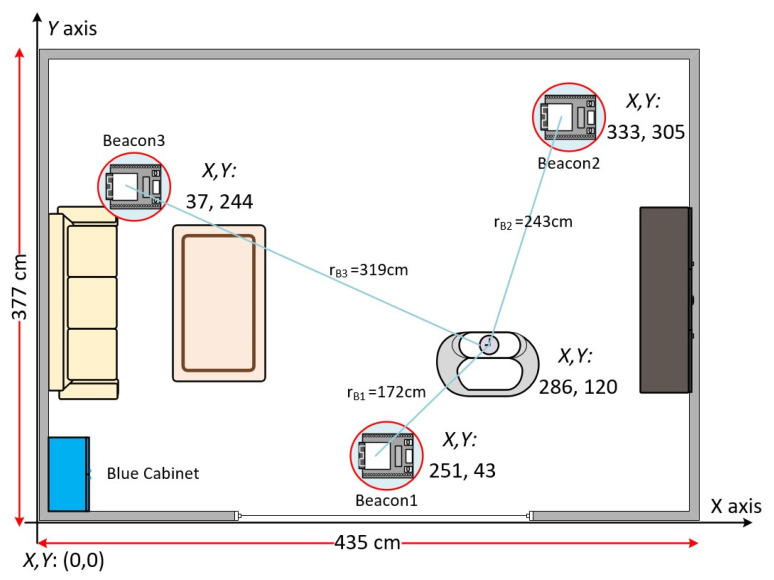
A prototype testing environment.

The testing environment is shown in Figure 4. The prototype test environment is a 12 m^2^ room with three beacons to test the signal transmission of these beacons in a small space. First, the origin of coordinates *X*, *Y* = (0, 0) the room is defined as the corner near the blue cabinet. Three beacons are deployed in this room and simulate the user wearing a smart bracelet for signal measurement. The three beacons are named Beacon 1, Beacon 2, and Beacon 3, which are located at coordinates (251, 43), (333, 305), and (37, 244), respectively. The data collected by these three beacons are sent to the Data Collection Server (DCS) for storage to detect the relative RSSI signal strength.

### 2.5. Positioning Method

In a general indoor positioning study, the system first analyzes the RSSI signal strength of the user device. It then calculates the straight-line distance as the basis for positioning based on the RSSI signal strength. However, in a realistic situation, the position of the smart bracelet may vary depending on the user’s behavior, such as standing or sitting. In this case, the user’s position in space remains the same, but the straight-line distance calculated from the RSSI signal strength is different, and therefore, positioning error may occur.

**Figure 5:**
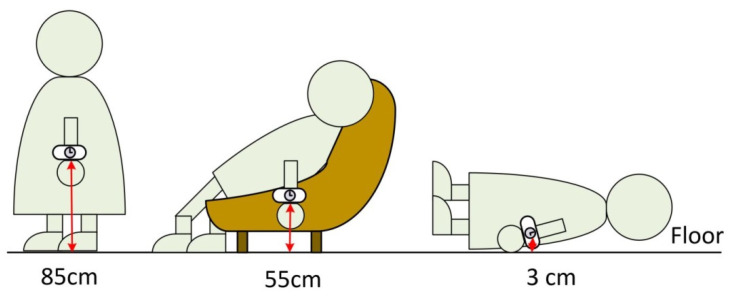
Three types of behavior.

The typical behaviors of people in space can be classified into three types: standing, sitting, and lying down. Generally, when an adult wears a smart bracelet, the heights of the smart bracelet under the above three behaviors can be defined as 85, 55, and 3 cm (shown in Figure 5).

**Figure 6:**
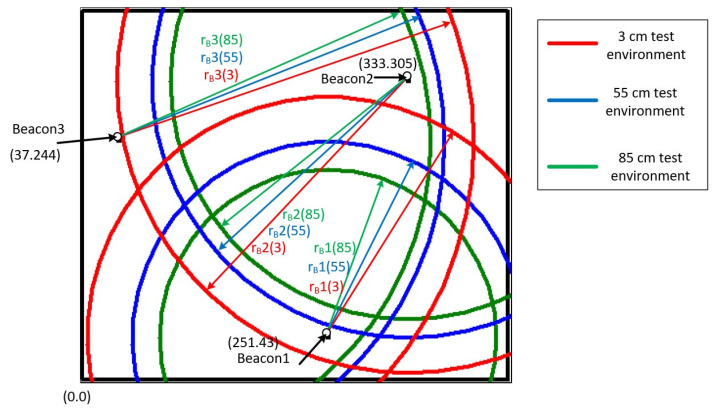
Distance to three coordinates for different beacons.

Figure 6 shows the RSSI signal strengths of the three beacons at different heights of the smart bracelet at the same location in the test environment. The concentric circles drawn with these three beacons as the center points are shown in green for 85 cm, blue for 55 cm, and red for 3 cm.

The values of r_B_i(85), r_B_i(55), and r_B_i(3) represent the signal radii of beacon i at heights of 85, 55, and 3 cm, respectively, where the values of i are 1, 2, and 3. According to the physical concept, these data should be mainly focused on the location of the bracelet. Therefore, this study analyzes the user’s position by calculating the vertical (v_B1_, v_B2_, v_B3_) and horizontal components (h_B1_, h_B2_, h_B3_).

**Figure 7:**
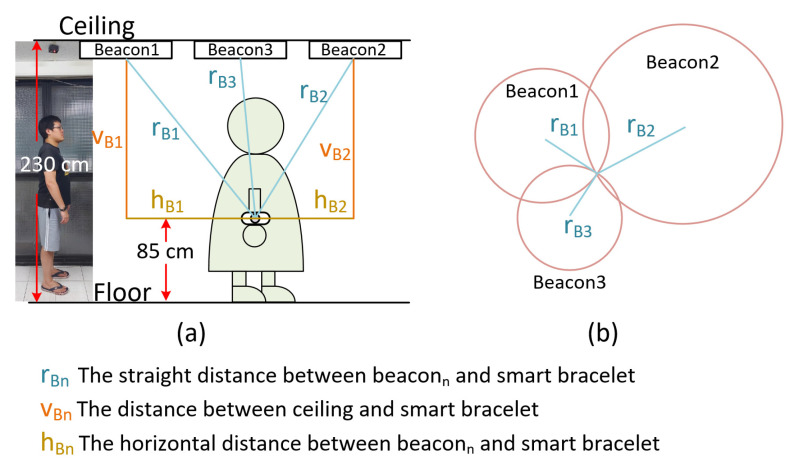
(**a**) A diagram of the calculation and the conversion of signal strength to horizontal distance. (**b**) The relationship between the bracelet and the three beacons in the standard triangulation system.

In a standard triangulation system, the position of the bracelet can be obtained from the signal strength between the three beacons, as shown by r_B_1, r_B_2, and r_B_3 in Figure 7b. Nonetheless, this is a two-dimensional signal in a plane, which means a straight-line distance between the beacon and the bracelet. In this study, the beacon is set on the ceiling to reduce the obstacles between the beacon and the bracelet and improve positioning accuracy. The horizontal distance of the bracelet in this space (h_B_1, h_B_2, and h_B_3) is then calculated using the Pythagorean theorem, as shown in Figure 7a.

### 2.6. Data Analysis Methods

First, the beacons and the bracelet are placed according to the positions shown in Figure 3, and their signals are obtained. Here, the RSSI received signal strength measurement method is a simple method of measuring distance because it will calculate the corresponding distance according to the weakening of the signal distance. The standard mathematical model equation is

(1) $$\mathrm{RSSI}=-\left(10nlog_{10}d+A\right)$$

**Figure 8:**
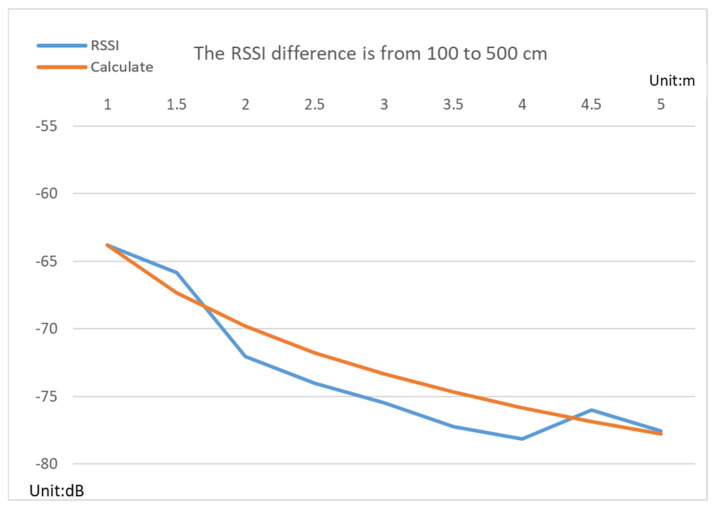
The RSSI difference is from 100 to 500 cm.

Here, *A* is the RSSI value received at a distance of 1 m between the sending and receiving sides. *n* is the path loss exponent, *d* is the distance between the sending and receiving sides, and the unit is meters. Therefore, a beacon was first started at a distance of 1 m and measured every 50 cm until a distance of 5 m, as shown in Figure 8. According to the rule of thumb, each beacon needs to go through this testing to obtain different *A* values. The data in Figure 8 suggest that the data obtained in the real space and the converted ideal value have a considerable error, but at least the measured results have almost the same decay conditions. Although other studies processed data through filters, which could improve the accuracy of RSSI distance measurement, they could not distinguish the height of the device.

**Figure 9:**
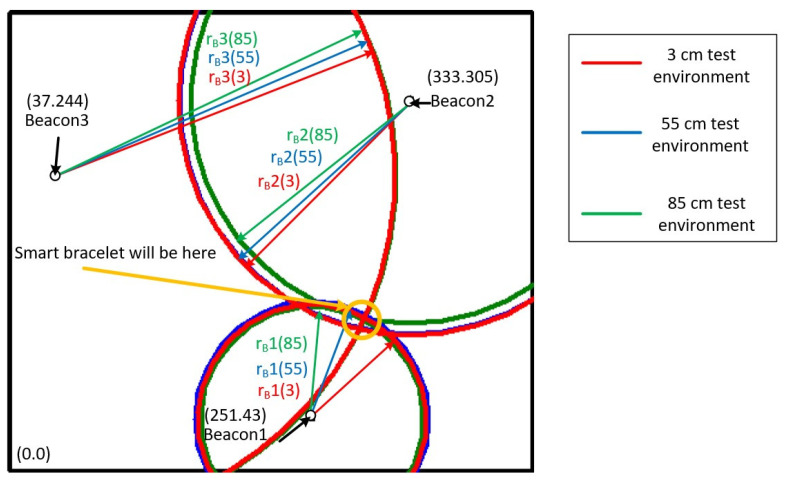
Predict the position of the smart bracelet.

Therefore, by reading the signal strength of at least three beacons, we can find the approximate position of the smart bracelet by using this method to calculate the vertical and horizontal vectors, regardless of the height of the smart bracelet, as shown in Figure 9. This approach will provide greater flexibility for monitoring the status of users quarantined at home or in hotels.

### 2.7. Emergency Recognition

The most common emergency for people living alone at home is a fall, and there are two main ways to detect a fall. The first method uses a camera to capture images and identify whether a fall has occurred through image analysis. The second way is to watch the sensor of the smart wearable device or smartphone by detecting the acceleration value of the device to determine whether the user has a momentary fall.

When using the camera to capture images, some areas are not suitable for use, such as bathrooms, toilets, or hotel rooms. The installation of cameras in these areas will cause users to feel disgusted and even infringe on the possibility of personal privacy. Analysis of the data measured by smart device sensors can effectively reduce user concerns about the invasion of personal privacy. However, some special situations cannot be determined by analysis of the data, such as when the user is sitting on the sofa, lying on the ground to sleep, etc.

This study uses Bluetooth beacons to position the user’s location by calculating vertical and horizontal vectors and is used for activity recognition. The relative behavior height of the user is obtained by analyzing the positioning data, and the measured data are sent back to the system for analysis through MQTT. Relevant emergency response procedures will be initiated if the analysis results show that the user may be in danger. For example, case 1: Under normal circumstances, the number of steps increases, and the heart rate stays average. Case 2: In the case of a fall, the number of steps did not increase, and the heart rate was slightly above average. At this point, the system will send a warning message to the caregiver’s smartphone set by the system, asking the caregiver to provide care. Case 3: During sleep, step count does not increase, and heart rate remains average, but blood oxygen level is checked because the user must pay special attention to his blood oxygen level (SpO2%) during home isolation. Sudden death caused by hypoxemia in solitary individuals during home isolation is not uncommon. The system will not only send a notice to the caregiver to care for the user but also continue to remind the caregiver of subsequent physiological conditions.

### 2.8. Emergency Notification Service

When the user may be in danger, the system communicates with different software through the INSTANT messaging module of the Node-Red management tool. This function is a one-way message notification, which only sends messages to the chat room of the instant messaging software commonly used in the market. The primary purpose of this function is to notify the healthcare unit that there is an emergency requiring immediate confirmation and exclusion or to notify designated personnel, such as family members or caregivers, that the monitored user needs help.

## 3. Results

**Figure 10:**
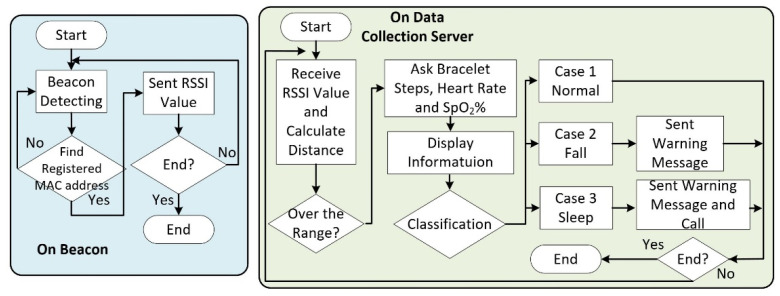
A flowchart of the low-cost indoor positioning system.

Before the system is executed, the MAC addresses of the smart bracelet and each beacon must be stored so that the system can recognize them. Figure 10 is a flow chart of this system; on the left side is the flow chart of each beacon. For each beacon, when the power is on, the beacon will start scanning all nearby Bluetooth devices. When a registered Bluetooth device is found, it will send the Bluetooth signal strength value back to the data collection server through wireless network transmission. The right side of Figure 10 shows the flow chart of the data collection process. When the system starts, it will receive the RSSI value from each beacon. At this time, the server will calculate the relative position between each beacon and the smart bracelet and will mark the position of the smart bracelet in this area.

**Figure 11:**
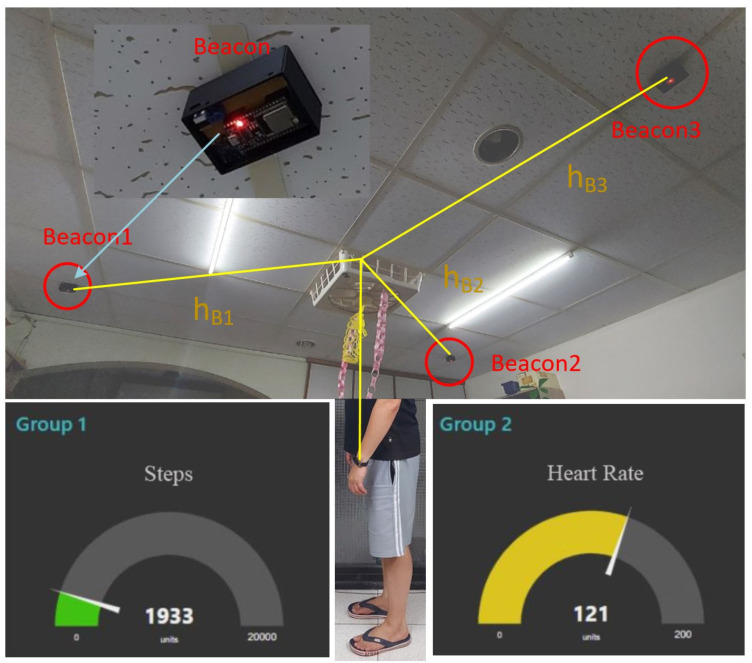
The position of each beacon device on the ceiling.

This study was conducted in the living room of the researcher’s home, where the equipment was directly built. Multiple Bluetooth beacons are placed on the ceiling for indoor positioning, as shown in Figure 11. User positions are analyzed by calculating vertical and horizontal components. Although the hardware device specifications of the Bluetooth signal are the same, comparing the analyzed data using the A value in Formula (1) measured by a group of Bluetooth beacons with the real distance data, there will be a small amount of error. Therefore, it is determined that each beacon needs to be tested to obtain different A values in Formula (1) before the data can be converted into distances. The data in Figure 11 show that although there is a considerable error between the data obtained in real space and the converted ideal value, at least the measured results have almost the same attenuation conditions. Almost every beacon measures this base value differently because variable A in Formula (1) is the signal strength measured at one meter. Different receiving devices do not produce the same results even in different spaces.

**Figure 12:**
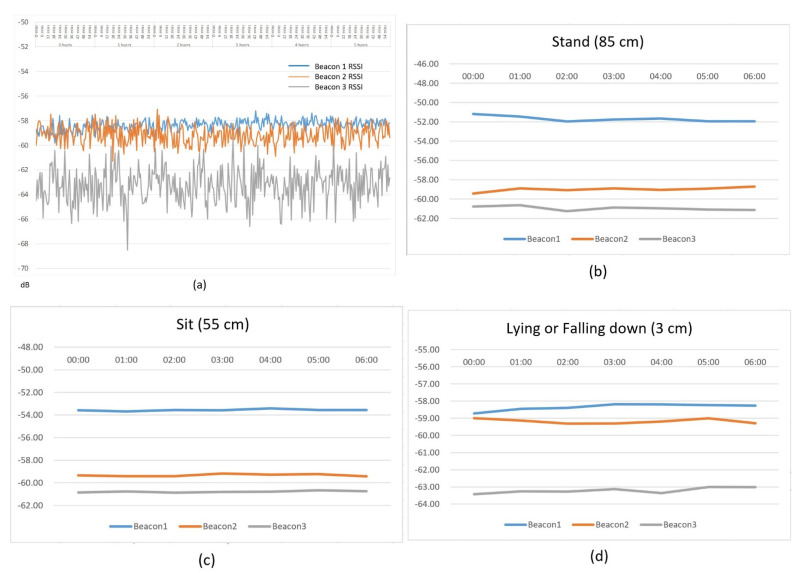
The signal strength of each beacon at three different heights. (**a**) The massive amount of data returned by the beacon. The average filter calculates the average arithmetic value of RSSI values received by nodes. The result represents in (**b**) 85 cm (**c**) 55 cm (**d**) 3 cm, respectively.

The smart bracelet was fixed at 85, 55, and 3 cm in this experiment. These positions represent standing, sitting, and lying down (falling). The data were measured between 12:00 am and 06:00 am to serve as a baseline for subsequent experiments. The measurement results are shown in Figure 12. Figure 12a shows the massive amount of data returned by the beacon. RSSI values received from Bluetooth devices can be corrupted due to a variety of factors, such as the presence of high-density obstacles and many indoor settings. Therefore, mean and median filters must be used to remove these outliers [42]. In this experiment, each beacon sends a signal strength value to the DCS, which takes an average of 5–8 s. To make the conversion distance more accurate, the mean filtering method is used to solve the problem of small signal differences effectively. The average filter calculates the average arithmetic value of RSSI values received by nodes. The results shown in Figure 12b–d represent the signal strength of the smart bracelet measured at three beacons of three different heights.

In this defined space, the coordinates of Beacon 1 are Beacon (X_b_1, Y_b_1), and the distance r_B_1 can be obtained from the signal strength data in Figure 12. r_B_2 and r_B_3 can also be obtained.

Table 2 shows the horizontal distances (h_B_n) obtained when each beacon reads three different heights (v_B_n). However, in real space, the data in Table 2 may not depict the real smart bracelet’s actual position in the area.

**Figure 13:**
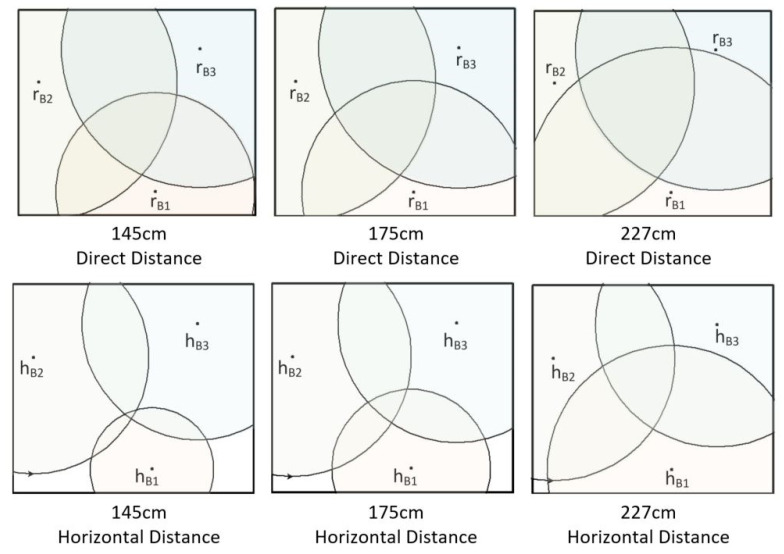
The result is when the direct distance is converted into a horizontal distance at three different heights.

The position is represented by a graph, as shown in Figure 13, which is the result of converting the direct distance to three horizontal distances at different heights (85, 55, and 3 cm). There is not necessarily an intersection, but you can get a predictable range from the intersection of three concentric circles, where the smart bracelet is likely to be.

### 3.1. Abnormal State Analysis

**Figure 14:**
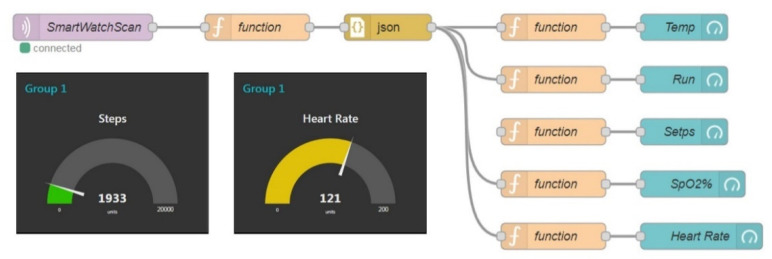
MQTT management procedure and remote physiological signal monitoring display screen.

Figure 14 shows the management and analysis process of physiological measurement data collected by the Node-RED management tool. When the smart bracelet receives the request of the data collector, it will perform the specified physiological measurement on the user, and the measured data will be uploaded to the data collector. The data uploaded to the data collector are decomposed after parsing. The heart rate, pace, and body temperature values are extracted, stored in the corresponding database, or provided with other applications. When new data are generated, such as a heart rate, the program is programmed to analyze whether the data are below standard. The regular heart rate of a healthy adult is approximately 60–80 beats per minute. Heart rates between 50 and 100 beats per minute are acceptable due to other environmental factors. When the data are less than 50 times or greater than 100 times, the user may have health problems and need emergency medical assistance. The system will send a notification message to the specified device.

### 3.2. Emergency Notification

**Figure 15:**
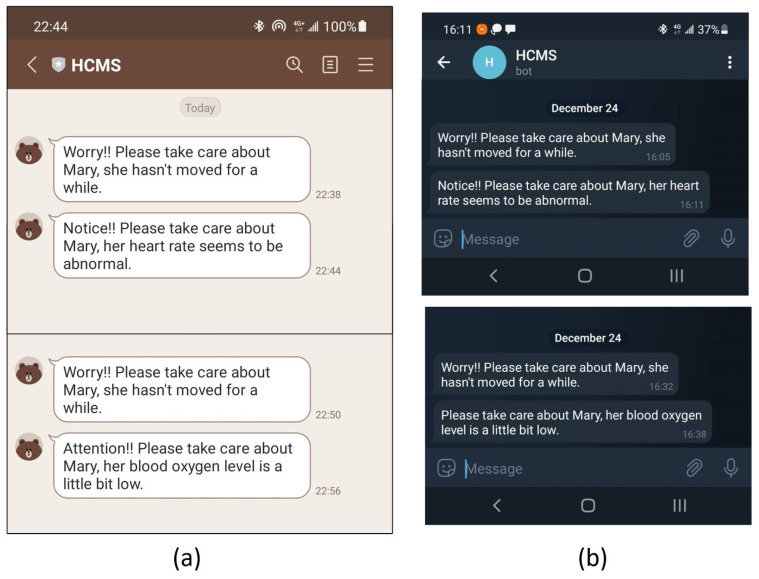
Display of the warning messages on the smartphone. (**a**) shows the notification screen when using Line, and (**b**) shows the notification screen on Telegrams.

Figure 15 shows a demonstration of emergency notification via instant messaging software. In the emergency notification process, the system sends a reminder notice first and then carries out the physiological measurement for a range of times. If the measured data do not meet the standard value, the system sends a warning message again. For example, the case in Figure 15 is the tester’s smart bracelet position, and the system identifies it as lying down. The first step value is stored first, and the step value is read again after a period of time. The first reminder notification is sent when the two values are equal or too close. shows in Figure 15 sentences that begin with “Worry”. Additional physiological measurements will be taken at intervals. A more dangerous warning message is issued if the equivalent measurement is too low. Figure 15a shows the notification screen when using Line, and Figure 15b shows the notification screen on Telegrams, another messaging software.

## 4. Discussion

From the experimental results in Figure 13, it can be seen that the user location can be roughly analyzed by calculating only the vertical and horizontal components of the indoor positioning method without using filtering for the measured data. It is also found that this study was tested in a real environment, where many factors, such as too much furniture or objects and signal interference from many electronic devices, can affect the positioning data, which can lead to deviations. Hardware specifications for beacons may be another possibility for improved accuracy, but more advanced equipment means higher overall equipment costs. Perhaps an increase in the number of beacons could improve positioning accuracy, which is expected to be the next experiment in this study.

The function of emergency recognition is to start the corresponding program by analyzing the location results. Although the results could not be pinned to a single seat, there was little difference in determining whether a person had fallen because this method also relied on the device’s height. In the experimental results in Figure 13, when the user is standing and the distance between the bracelet and the ceiling is 145 cm, the bracelet is the closest to the beacon device, and the data error is the smallest. When the user is sitting down or lying down and the distance between the bracelet and the ceiling is 175 and 227 cm, the data error gradually increases. Therefore, we can think of this situation as a potentially hazardous situation for the user. In this case, the system will put forward physiological measurement requirements for the smart bracelet.

The primary purpose of the smart bracelet is to provide Bluetooth beacons to read the signal strength for indoor positioning and provide physiological measurements for behavioral analysis. Although the self-designed smart bracelet offers a high degree of integration, the device’s function, size, and endurance cannot meet the expected standards. Other modular or commercialized smart bracelets are also used during the experiment.

Table 3 shows the comparison with other research. The low-power indoor positioning system proposed in this study can be applied to situations where home isolation is required. The beacon device using an embedded microprocessor can achieve the same positioning effect as the micro single-board computer based on the ARM architecture with lower-cost hardware specifications. In addition, the small beacon device also has a quick disassembly function. Micro single-board computers provide data security with the same capabilities as small servers and private cloud system architecture.

## 5. Conclusions

In recent years, with the rapid spread of the COVID-19 epidemic around the world, countries have designated specific places (such as homes or hotels) where people entering the country must be quarantined for a certain period of time. Most quarantine spaces install sensors at the door and use the smartphone with nearby mobile phone base stations to determine if the guest is staying in the hotel. However, during the isolation period, it must be possible to ensure that the person is not in contact with others while staying in the room and determine whether he is in good health. Medical personnel or control personnel must call or send a text message within a fixed period to determine the person’s health. These preventive activities will consume considerable manpower and time, which is the relative waste of many resources. The design of this study can effectively reduce the waste of resources by using Bluetooth beacons, a traveler detection smart bracelet suitable for small spaces, to ensure that people are staying in the room. Through the physiological sensing signal of the smart bracelet, it can determine whether people are still staying indoors, and detecting heart rate in the physiological signal can ensure that they are living in a more reasonable state. If there are abnormal data or behaviors, the system will notify the medical staff for care through instant messaging software so that it will not affect the living habits of passengers and the workload of medical staff. Finally, due to the advantages of rapid installation and low cost, the system can be adjusted in each epidemic prevention space without a particular monitoring environment for each epidemic prevention space.

After many experiments, we came to several conclusions. (1) The RSSI signal is volatile, especially when someone passes by, metal devices are in the measurement range, or electrical signals are generated; there will be data changes. (2) This paper converted problematic RSSI signals into trigger signals to read physiological information from smart wristbands. When drift occurs, the user’s physiological information will be read. The traditional way is to read it at fixed times, but accidents often happen at varying times. If the system continues to read information about the smart bracelet, it is easy to reduce the standby time of the smart bracelet. In this way, these problems can be solved by minimizing privacy to determine whether a solitary person stays at home. (3) There is no problem with the reading signal strength of the smart wristbands on the market. However, if users want to read the number of steps, heart rate, and blood oxygen level and these data cannot be easily read due to personal privacy, they must use the self-designed smart wristband to achieve this function. In the future, different algorithms, such as Gaussian filters or Kalman filters, will be used to gain more accurate positioning. Second, this paper is the hardware equipment basis for human space behavior prediction. Through the analysis and collection of these data, it is hoped that it can predict human space behavior such as standing up, sitting down, and lying down without using the photographic lens. Finally, it is expected that the prediction of human behavior can be achieved without smart wristbands.

## Figures and Tables

**Table 1:** Comparison table of common indoor positioning technologies and their advantages and disadvantages.

Positioning Method	Advantages	Disadvantages
RFID [25,26]	The error of location information is small. The signal transmission range is extensive, and the cost is low.	Small positioning range. No communication ability. Poor anti-interference ability. User security, privacy protection, and international standardization are not perfect.
Wi-Fi [27,28]	They provide large-scale positioning, monitoring, and tracking tasks. Shared networks. Low hardware cost and Wi-Fi positioning systems reduce the probability of radio frequency (RF) interference.	Transmitted signals are easily interfered with by noisy signals, thus reducing positioning accuracy. To achieve indoor positioning, multiple access points are required, and therefore not suitable for general household use.
Ultra wideband (UWB) [29,30]	High penetrability, low power consumption, little influence by multipath effect, high safety factor, lower system complexity, and high positioning accuracy.	UWB positioning requires special equipment as its time and pulse requirements are more accurate, so the price is high.
Ultrasonic [31,32]	Ultrasonic positioning accuracy is very high.	The transmission rate of the sound wave is relatively low, and it is difficult to overcome the multipath and non-line-of-sight effects. The overall cost is high, and it is not easy to maintain in the future.
ZigBee [33,34]	It provides short-range and low-rate wireless network technology, low power consumption, and high efficiency.	Signal transmission is greatly affected by the multipath effect and movement, and the cost of positioning software is high.
Infrared [35,36]	Low cost and good positioning effect.	Infrared transmission distance is short and cannot go over obstacles. The total cost is high because receiving antennas must be installed in every room and corridor to improve accuracy.
Bluetooth [37,38]	It is a wireless transmission technology that can be applied for small-distance positioning. In addition, the device size is small, so it is not affected by the line of sight.	In the testing environment, the corresponding Bluetooth area network access point should be arranged in advance, and then the corresponding network connection mode should be configured so that the accuracy can be kept within 3~15 m.

**Table 2:** The relative distance from each beacon to the smart bracelet.

	Distance	145 cm	175 cm	227 cm
Beacon 1	RSSI	−51.7 dB	−53.56 dB	−58.35 dB
	r_B1_	183 cm	203.7 cm	268.4 cm
	h_B1_	111.7 cm	143.2 cm	225.8 cm
Beacon 2	RSSI	−59.0 dB	−59.33 dB	−66.9 dB
	r_B2_	254.1 cm	259.0 cm	256.7 cm
	h_B2_	208.7 cm	24.6 cm	211.8 cm
Beacon 3	RSSI	−60.9 dB	−60.8 dB	−63.2 dB
	r_B3_	324.7 cm	321.5 cm	369.6 cm
	h_B3_	290.6 cm	286.9 cm	340.0 cm

The height of v_B_n is calculated as the ceiling height (230 cm) minus the smart bracelet height of 85, 55, and 3 cm.

**Table 3:** Comparison table of price and power consumed when using different positioning sensing devices.

	Main Positioning Sensing Device	Consumed Power	Equipment Price	Quantity Required
Research 1 [27,28]	Wi-Fi Access Point	12 V/1 A/12 W	USD 41.35	At least three
Research 2	Smart Camera	5 V/2 A/10 W	USD 37.57	At least one
Research 3 [4]	Raspberry Pi	5 V/0.5 A/2.5 W	USD 101.93	At least three
This research	ESP32 Micro Controller	5 V/0.26 A/1.3 W	USD 7.12	At least three

Refer to the Amazon^®^ Official Site for power consumption and pricing for each device.

## Data Availability

Not applicable.
